# The History of Plague

**Published:** 1902-11-08

**Authors:** 


### THE HISTORY OF PLAGUE.

THE continuation of the history of plague throughout the world, from the middle of 1898 to the middle of 1901, which has been prepared by Dr. Bruce Low, and published by the Local Government Board as a supplement to the report of the medical officer, is not only a document of the highest interest and importance to epidemiologists, but it is also one which is well calculated to allay any anxieties which may have been felt concerning the probability of an invasion of Europe by the disease. Except in some Oriental countries, and notably in India, one principal result of Dr. Low's inquiries, as summarised by Mr. Power, F.R.S., in his general introduction to the history, has been to show that plague "has manifested only small ability to become seriously epidemic;" and he infers that it is far inferior to cholera in its ability to become acclimatised to conditions other than those obtaining in its own endemic area. The conditions as to receptivity of this country have manifestly undergone complete alteration since the great pestilence of the seventeenth century; but it seems not to be impossible that the virulence of the imported bacillus may also have undergone diminution, contemporaneously, and possibly in some connection with, a corresponding diminution of its power of maintaining a saprophytic existence. Nothing is more curious than the way in which the first cases of the disease, in many localities, have presented deceptive resemblances to maladies of common occurrence among the inhabitants, such as influenza, enteric fever, and even malarial fever; resemblances which have deceived even those who were on the look out for plague, and who thought themselves prepared to recognise the earliest evidence of its occurrence. From this cause, Dr. Low tells us, it has frequently been impossible, when the disease could no longer be overlooked or mistaken, to obtain any certainty as to the precise date of its invasion, or even as to the channels through which it had been introduced. By the time when its presence was definitely established by bacteriological or clinical evidence, the precise nature of suspicious cases of antecedent illness among groups related socially or in other ways to the subsequently declared plague cases had usually become matter of surmise.

Upon the whole, and assuming the facts to be correctly stated, it would seem probable that an explanation of them may ultimately be afforded by the attainment of more exact knowledge than is now possessed with regard to the influence of environment upon many forms of pathogenetic bacilli; for it is clear that some, at least, of these vary in their activity in a manner that is not fully understood. Professor Delépine, to whose many and great accomplishments a sense of humour does not appear to be superadded, has lately informed the world that certain deaths from eating unwholesome pork pie were due to the presence therein of the *bacillus interitidis Derbyensis*, as if a microbe had been specially created for the use of the citizens of the important midland town concerned. The bacillus of plague as it exists in Bombay may present similar differences from the bacillus of plague as it becomes modified by emigration to Glasgow or to Cardiff. The probability of such modification is further enhanced by the difference of the fate which befell rats in the two last-named places. In Glasgow the rats appear to have escaped entirely, although 36 cases and 16 deaths occurred in human subjects in the months of August and September, 1900. In Cardiff the mortality among rats was large, and was distinctly traceable to the plague bacillus, but only one man was attacked. Nor is apparent identity with difference of action limited to the organisms which fall under the observation of the bacteriologist. It occurs equally among those to which a place in the animal kingdom is assigned. The parasite communicated to horses by the tsetse fly, and which is rapidly fatal to them, is apparently identical with one which is common, and apparently harmless, in the blood of the rat; while the rat is quickly killed by an injection of infected blood from a tsetse bitten horse. Within the last few days, moreover, a parasite resembling those of the rat and of the horse, but possibly possessing qualities different from either, has been discovered at the London School of Tropical Medicine in the blood of a woman suffering from a peculiar type of chronic fever, with splenic enlargement and cutaneous erythema, which has for some time been known as resembling malarial fever and yet differing from it, as resisting quinine, and as tending towards a fatal issue. The new parasite, we are told, swims freely in the blood serum; and, if it should hereafter be proved to be the cause of the disease with which it is associated, the discovery seems to hold out reasonable hope of the attainment of a means of cure. In the meanwhile its presence, together with its apparently variable behaviour in different circumstances, and with the variable behaviour of the plague bacillus, seem to show that we still have much to learn with regard to the operation of agencies with whose outward aspects we have recently become familar.

